# Multilevel Laminoplasty for CSM: Is C3 Laminectomy Better Than C3 Laminoplasty at the Superior Vertebra?

**DOI:** 10.3390/jcm12247594

**Published:** 2023-12-09

**Authors:** Mohamed Macki, Timothy Chryssikos, Seth M. Meade, Alexander A. Aabedi, Vijay Letchuman, Vardhaan Ambati, Nishanth Krishnan, Michael E. Tawil, Seth Tichelaar, Joshua Rivera, Andrew K. Chan, Lee A. Tan, Dean Chou, Praveen Mummaneni

**Affiliations:** 1 Cleveland Clinic Center for Spine Health, Cleveland Clinic Foundation, Cleveland, OH 44195, USA; 2 Department of Neurological Surgery, University of California, San Francisco, CA 94143, USApraveen.mummaneni@ucsf.edu (P.M.); 3 Cleveland Clinic Lerner College of Medicine, Cleveland Clinic Foundation, Cleveland, OH 44195, USA; 4 University of California San Francisco Medical School, University of California San Francisco, San Francisco, CA 94143, USA; 5 Department of Neurosurgery, Stanford University, Stanford, CA 94305, USA

**Keywords:** cervical, laminectomy, laminoplasty, lordosis

## Abstract

Introduction: In a multilevel cervical laminoplasty operation for patients with cervical spondylotic myelopathy (CSM), a partial or complete C3 laminectomy may be performed at the upper level instead of a C3 plated laminoplasty. It is unknown whether C3 technique above the laminoplasty affects loss of cervical lordosis or range of motion. Methods: Patients undergoing multilevel laminoplasty of the cervical spine (C3–C6/C7) at a single institution were retrospectively reviewed. Patients were divided into two cohorts based on surgical technique at C3: C3–C6/C7 plated laminoplasty (“C3 laminoplasty only”, N = 61), C3 partial or complete laminectomy, plus C4–C6/C7 plated laminoplasty (N = 39). All patients had at least 1-year postoperative X-ray treatment. Results: Of 100 total patients, C3 laminoplasty and C3 laminectomy were equivalent in all demographic data, except for age (66.4 vs. 59.4 years, *p* = 0.012). None of the preoperative radiographic parameters differed between the C3 laminoplasty and C3 laminectomy cohorts: cervical lordosis (13.1° vs. 11.1°, *p* = 0.259), T1 slope (32.9° vs. 29.2°, *p* = 0.072), T1 slope–cervical lordosis (19.8° vs. 18.6°, *p* = 0.485), or cervical sagittal vertical axis (3.1 cm vs. 2.7 cm, *p* = 0.193). None of the postoperative radiographic parameters differed between the C3 laminoplasty and C3 laminectomy cohorts: cervical lordosis (9.4° vs. 11.2°, *p* = 0.369), T1 slope–cervical lordosis (21.7° vs. 18.1°, *p* = 0.126), to cervical sagittal vertical axis (3.3 cm vs. 3.6 cm, *p* = 0.479). In the total cohort, 31% had loss of cervical lordosis >5°. Loss of lordosis reached 5–10° (mild change) in 13% of patients and >10° (moderate change) in 18% of patients. C3 laminoplasty and C3 laminectomy cohorts did not differ with respect to no change (<5°: 65.6% vs. 74.3%, respectively), mild change (5–10°: 14.8% vs. 10.3%), and moderate change (>10°: 19.7% vs. 15.4%) in cervical lordosis, *p* = 0.644. When controlling for age, ordinal regression showed that surgical technique at C3 did not increase the odds of postoperative loss of cervical lordosis. C3 laminectomy versus C3 laminoplasty did not differ in the postoperative range of motion on cervical flexion–extension X-rays (23.9° vs. 21.7°, *p* = 0.451, N = 91). Conclusion: There was no difference in postoperative loss of cervical lordosis or postoperative range of motion in patients who underwent either C3–C6/C7 plated laminoplasty or C3 laminectomy plus C4–C6/C7 plated laminoplasty.

## 1. Introduction

Since its inception in 1977 by Hirabayashi [1], expansive open-door cervical laminoplasty has been traditionally performed for the treatment of cervical myelopathy in patients who do not have cervical kyphosis, severe axial neck pain, or predominantly radicular symptoms [2]. By reconstructing rather than removing the cervical lamina in its entirety [3], laminoplasty provides the superior preservation of preoperative lordosis to simple laminectomy [4,5] and results in an increased range of motion [6] at lower total cost compared with posterior cervical fusion [7]. Nevertheless, loss of cervical lordosis can occur following laminoplasty. Because the preservation of cervical lordosis after laminoplasty is one of the primary goals of surgery, and because greater cervical lordosis is associated with better pain outcomes [8], awareness of the factors that predict loss of cervical lordosis following laminoplasty is of continuing interest to spine surgeons.

The relationships between preoperative radiographic parameters and loss of cervical lordosis following laminoplasty have been fairly well studied. Several studies have concluded that a high T1 slope and the difference between T1 slope and C2–C7 lordosis can predict the loss of lordosis following laminoplasty [9,10,11,12,13,14,15,16,17,18]. The relationship between loss of cervical lordosis following laminoplasty and spinal parameters beyond the regional cervical spine on full-length scoliosis X-rays (i.e., pelvic incidence, lumbar lordosis, sagittal vertical axis from C7–S1) have been understudied by comparison. Although several studies have concluded that preoperative dysfunction of the paraspinal musculature or diminished muscle mass may predict loss of lordosis [19,20,21], these studies have been contradicted by a recent study of the cross-sectional area of the deep extensor muscles which found no relationship with loss of lordosis [20]. Interestingly, one study reported a 120% recovery of neck muscle strength at 12 months in laminoplasty patients without preoperative neck pain, compared with 60% in patients with preoperative neck pain. Finally, the relationship between loss of lordosis and clinical outcome, as determined via patient-reported outcome measures, remains relatively understudied, and the results that are currently available have been mixed [19,22,23,24,25,26].

Several studies have investigated the relationship between loss of lordosis and surgical technique. With regard to instrumentation, mini-plate fixation has shown superior clinical and radiographic outcomes compared with suture suspension [27] and shorter operative times compared with structural allografts [28]. Attention has also been directed toward the selective preservation of muscular and ligamentous integrity during laminoplasty. Several studies have advocated for semispinalis cervicis repair and the preservation of the muscular attachments to the C2 spinous area to prevent postoperative kyphosis [25,29,30,31]. In contrast, other studies have concluded that the preservation of the subaxial deep extensor muscles plays no role in preventing loss of lordosis following laminoplasty, but rather it is preservation of the muscular attachments to the C2 and C7 spinous processes specifically that prevents postoperative kyphosis [30,32].

It has been further suggested that the preservation of the ligamentum flavum between C2 and C3 results in better postoperative lordosis after laminoplasty [30]. The importance of this ligament for postoperative stability finds its anatomic correlate in a cadaveric study by Panjabi et al., who reported an increased mean width of the ligamentum flavum at C2–3 and shorter length of interspinous ligament at C2–3 compared with the rest of the subaxial cervical spine [33]. One study compared laminoplasty starting at either C3 or C4 and reported significantly less loss of lordosis in the C4 group [34]. When using skip-level plating, another group reported improved postoperative lordosis when plating at C4 and C6 rather than C3 and C5 [35]. Finally, another group reported good clinical outcomes using partial undercutting (“dome”) laminectomy at C3 above C4 laminoplasty instead of C3 laminoplasty, but their study did not include a comparison group [36].

In this study, we sought to directly compare the impact of surgical technique at the C3 level: C3 laminoplasty vs. C3 partial “dome” laminectomy.

## 2. Materials & Methods

### 2.1. Study Design, Setting, Participants

All patients undergoing laminoplasty of the subaxial cervical spine (C3–C7) were retrospectively reviewed at a single institution from March 2009 to December 2019. Data were collected from electronic medical records and medical image databases (Epic Systems Corporation, Verona, WI, USA). Operations that included the occiput, C1, C2, or thoracic spine were excluded. Similarly, patients undergoing laminectomy at C2, C5, C6, T1, and T2, in addition to laminoplasty of the subaxial cervical spine, were excluded. All cervical fusions were excluded. Postoperative imaging was obtained over a median of 24.2 months. 

### 2.2. Variables

All patients underwent open-door laminoplasty. During exposure, the facet capsules were exposed bilaterally, using blunt dissection rather than electrocautery, for careful preservation. For each skeletal segment undergoing laminoplasty, a high-speed drill with a 3 mm matchstick burr was used to create a complete trough on one side between the lamina medially and the lateral mass laterally. On the contralateral side, a partial thickness trough was created via the preservation of the inner cortex of the lamina. The spinolaminar complex was then elevated upward toward the partial thickness (hinge) side. During the elevation, the ligamentum flavum was resected on the side of the complete laminar trough, and a laminoplasty plating system was secured between the lamina and lateral mass using screws.

The study population was divided into two cohorts: patients who underwent C3 laminoplasty, versus patients who underwent C3 partial “dome” laminectomy at the rostral decompression level. Undercutting (or “dome”) laminectomy was performed at the caudal end of the C3 spinolaminar complex using a high-speed drill with a 3 mm matchstick burr and Kerrison rongeur. The technique preserved the posterior tension band, including ligamentous and muscular attachments to the spinous process of the C2–C3 segment.

Radiographic measurements conducted on both preoperative and postoperative upright neutral X-rays of the cervical spine included C2–C7 lordosis, T1 slope, and cervical sagittal vertical axis. Lumbar lordosis, pelvic incidence, sacral slope, pelvic tilt, thoracic kyphosis, and global sagittal vertical axis (using C7 plumb line) were measured using standing long-cassette lateral scoliosis X-rays. Each of these measurements was defined as follows:

C2–C7 Lordosis: the angle formed between the parallel projections of the lower endplates of C2 and C7;

T1 Slope: the angle between the upper endplate of T1 and the horizon;

Cervical–Sagittal Vertical Axis (cSVA): this was determined by drawing a plumb line from the centroid of C2 to the posterior superior corner of C7;

Lumbar Lordosis: the angle between the parallel projections of the upper endplate of L1 and the upper endplate of S1;

Sacral Slope (SS): the angle between the sacral plate and the horizon;

Pelvic Tilt (PT): the angle between the line connecting the midpoint of the sacral plate to the axis of the femoral heads and a vertical line;

Pelvic Incidence (PI): the sum of sacral slope and pelvic tilt;

Thoracic Kyphosis: the angle between the upper endplate of T4 and the lower endplate of T12;

Global Sagittal Vertical Axis (GSVA): using the C7 plumb line, which is a vertical line drawn from the centroid of C7, the distance to the posterior superior aspect of the sacrum was measured.

Aside from surgical technique and radiographic measurements, other variables that could affect study outcomes were collected and analyzed, including age, sex, height, weight, body mass index, and comorbidities. Comorbidities were recorded, including smoking status, diabetes, coronary artery disease, peripheral vascular disease, chronic kidney disease, and osteoporosis. American Society of Anesthesiologists (ASA) classification was used a surrogate marker of comorbidity burden.

### 2.3. Primary Outcome

The primary outcome measure of this study was change in cervical lordosis, which was measured on lateral upright X-rays:

Δ cervical lordosis = preoperative cervical lordosis − postoperative lordosis.

A greater delta value equates to a more dramatic loss of cervical lordosis. Cervical lordosis was defined as the Cobb angle from the inferior endplate of C2 to the inferior endplate of C7.

### 2.4. Quantitative Variables

To account for variable differences in the change in cervical lordosis, the primary outcome measure was divided into three cohorts. A difference in cervical lordosis < 5° was defined as “no change”. A decrease in cervical lordosis between 5° and 10° was defined as “mild change”. A loss in cervical lordosis greater than 10° was defined as “moderate change”.

### 2.5. Statistical Methods

The laminoplasty patient population was described using summary statistics. Binary outcomes among the C3 laminoplasty and C3 partial “dome” laminectomy cohorts were compared using the chi-squared test. Continuous variables between the two cohorts were analyzed using a variance of ratio test. If the F statistic was not statistically significant, then a Student’s *t*-test compared the two cohorts. If the F statistic was statistically significant, then the analysis of variance was unequal and the comparison proceeded with Welch’s *t*-test.

The two-way measure of associations between the ordinal primary outcome—no change, mild change, or moderate change in cervical lordosis—and the prognostic factor of interest—C3 laminoplasty versus C3 partial “dome” laminectomy—was calculated using simple ordinal regression analysis. Other covariables were included in a multiple ordinal regression, reporting adjusted odds ratios (OR_adj_). The final regression equation was developed with forward stepwise modeling based on statistically significant variables in the univariable analysis.

## 3. Results

### 3.1. Descriptive Data

The 100 laminoplasty patients were similarly divided between laminoplasty at C3 (N = 61) and partial “dome” laminectomy at C3 (N = 39). With respect to demographic and prognostic factors in Table 1, the cohorts were similar with minimal exceptions. The C3 laminoplasty group had an older mean age of 66.4 years, while the mean age of the laminoplasty with partial C3 “dome” laminectomy group was 59.4 years (*p* = 0.012).

Median follow-up reached 26.5 months [range: 12.2 months–9.0 years] in the laminoplasty-only cohort and 15.3 months [15.3 months–6.4 years] in the laminoplasty with partial C3 “dome” laminectomy cohort (*p* = 0.002).

### 3.2. Outcome Data

The C3 laminoplasty cohort and the partial C3 “dome” laminectomy cohort did not significantly differ in preoperative cervical and global spinopelvic parameters (Table 2). The postoperative radiographic parameters also did not significantly differ.

Only one patient in the partial C3 “dome” laminectomy cohort (one-year reoperation = 2.5%) required a C5–C6 and C6–C7 anterior cervical discectomy and fusion for post-laminoplasty kyphosis four months after the index operation; no such occurrences were observed in the C3 laminoplasty cohort. Wound washout reached 6.5% in the C3 laminectomy cohort versus 2.5% in the C3 laminoplasty cohort (*p* = 0.371). Three patients had readmissions: one patient (1.6%) after C3 laminoplasty and two patients (5.1%) after partial C3 “dome” laminectomy (*p* = 0.318). Five days after discharge for C3–C6 laminoplasty, a 59-year-old male developed right-sided weakness with slurring of his speech. MRI brain and C-spine examinations were negative for strokes, and EEG was negative for seizures. Etiology was attributed to baclofen dosing, which was decreased. Twelve days after discharge, a 67-year-old female re-presented with acute onset left-eye vision loss, attributable to a relapse in primary central nervous system lymphoma. The patient was immediately restarted on chemotherapy. Twenty-six days after discharge, a 67-year-old re-presented with bilateral lower-extremity swelling, secondary to extensive bilateral deep venous thromboses that extended up to a long-standing inferior vena cava (IVC) filter. Interventional radiology was used to perform a thrombectomy with IVC retrieval.

### 3.3. Main Result

In total, 31% of patients (n = 31) had loss of cervical lordosis >5°. Loss of lordosis reached 5–10° (mild change) in 13% of patients (n = 13) and >10° (moderate change) in 18% of patients (n = 18). When stratified by surgical technique, there were no significant differences between the two cohorts (C3 laminoplasty versus C3 partial “dome” laminectomy) for loss of cervical lordosis.

In Table 3, an ordinal regression controlled for the only variable that was statistically significant in the univariable analysis: age. The primary outcome measure in the regression model was divided into three categories: no change (<5°), mild change (5–10°), and moderate change (>10°) in loss of cervical lordosis. For the prognostic variable of interest, neither of the surgical techniques—C3 laminoplasty or partial C3 “dome” laminectomy—predicted loss of lordosis following laminoplasty [OR_adj_ = 0.72, *p* = 0.479].

## 4. Discussion

The present study is part of an ongoing effort to understand loss of lordosis following cervical laminoplasty. Although much attention has been dedicated to the preoperative cervical spine radiographic parameters that predict loss of cervical lordosis, less attention has been dedicated to surgical technique, specifically, the surgical technique employed at the rostral segment of cervical laminoplasty.

**Figure 1:**
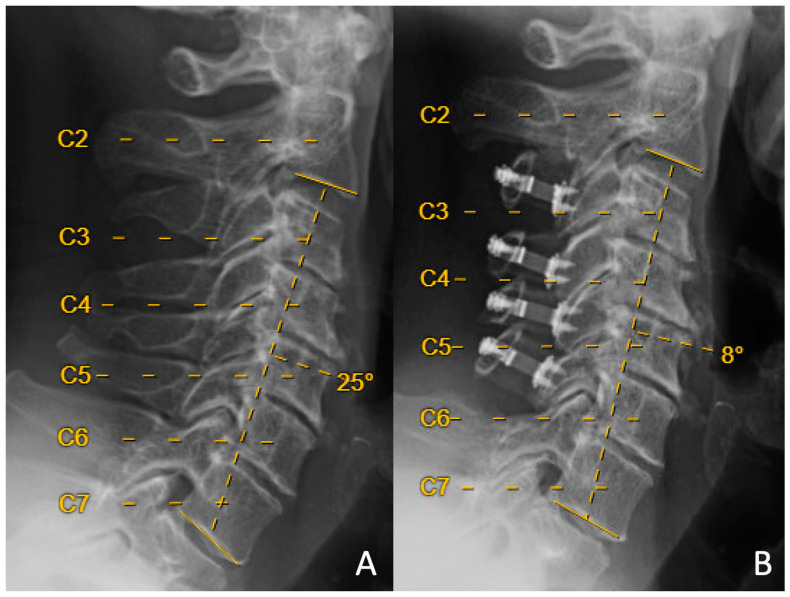
Preoperative (**A**) and 14-month-postoperative (**B**) lateral upright neutral cervical spine X-rays from a patient who underwent C3–C6 laminoplasty. Preoperative C2–C7 lordosis was +25 degrees, and postoperative C2–C7 lordosis was +8 degrees, with resulting loss of cervical lordosis of 17 degrees.

**Figure 2:**
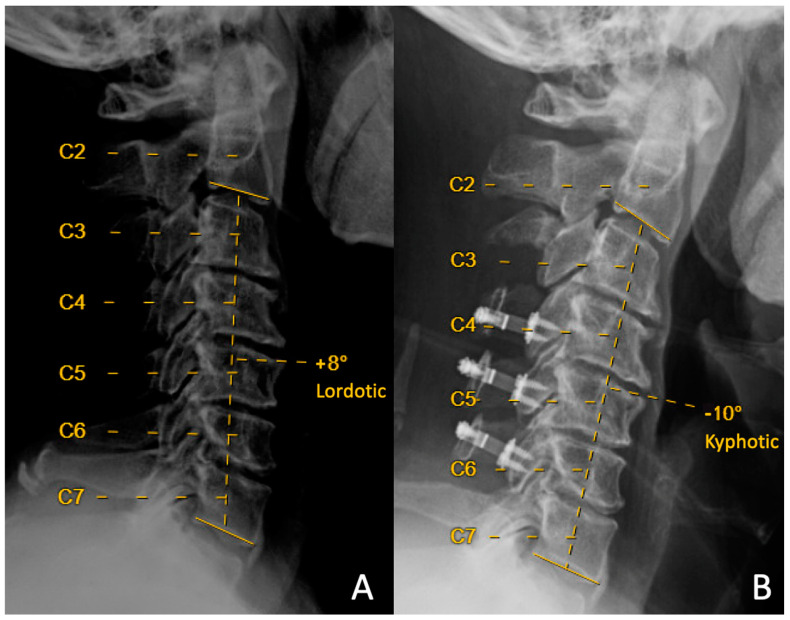
Preoperative (**A**) and 14-month-postoperative (**B**) lateral upright neutral cervical spine X-rays from a patient that underwent C3 partial laminectomy with C4–C6 laminoplasty. Preoperative C2–C7 Cobb angle was +8 degrees of lordosis, and postoperative C2–C7 Cobb angle was −10 degrees of kyphosis, with resulting loss of cervical lordosis of 18 degrees.

Here, we evaluated loss of lordosis in two cohorts, each with a unique surgical technique at the C3 level. Loss of lordosis was seen in patients who underwent both laminoplasty at C3 (Figure 1) and partial (“dome”) laminectomy at C3 (Figure 2).

It has been suggested that preservation of muscular and ligamentous attachments at the rostral end of the laminoplasty bed leads to preserved lordosis. Michael et al. [34] retrospectively studied 65 patients who underwent laminoplasty starting either at C3 (49 patients) or C4 (16 patients) and reported significantly less loss of lordosis in the latter group. However, this study did not directly compare surgical technique at the C3 level. An additional suggestion is that performing undercutting “dome” laminectomy at C3 rather than laminoplasty provides superior muscular and ligamentous preservation at the rostral end of the laminoplasty. Liu et al. [36] reported decreased loss of lordosis when employing C3 partial “dome” laminectomy. However, only 26 patients were included in this study, which did not include a comparison group. Umeda et al. [37] also reported preserved lordosis in 44 patients undergoing partial C3 laminectomy at two years follow-up, but this study also lacked a comparison group at C3. Lastly, instead of partial C3 laminectomy, Chen et al. [38] compared laminoplasty plus “standard” C3 laminectomy (37 patients, of whom an unspecified number also underwent C2 “dome-like expansive laminoplasty”) with laminoplasty only (74 patients). Contrary to what might be expected following standard laminectomy, these authors reported superior postoperative lordosis compared with laminoplasty only. The present study therefore represents the largest cohort study directly comparing C3 partial (“dome”) laminectomy with C3 laminoplasty. In the present cohort, multivariable analysis revealed that there were no significant differences between C3 laminoplasty versus C3 partial laminectomy.

An additional consideration for the discussion of surgical technique at the C3 level, beyond loss of lordosis, is postoperative pain. Significant preoperative neck pain has historically indicated cervical fusion rather than laminoplasty, but a study carried out by Mesfin et al. [39] raises questions about whether postoperative pain outcomes may vary by segmental level of laminoplasty. These authors reported significant improvement in minimum one-year postoperative NDI pain scores in 34 patients undergoing laminoplasty, of whom 24 (71%) did not have laminoplasty at C3 or C7. Riew et al. [40] also carried out a systematic review of 11 studies to determine whether preserving paraspinal muscle attachments at C2 and C7 leads to reduced postoperative pain following laminoplasty. Although the authors found conflicting results for both C2 and C7, they concluded that, so long as complete decompression is not required at C2 or C7, there is little reason not to preserve the paraspinal muscle attachments at these segments.

If the muscle attachments at C2 and C7 are the most crucial structures for preserving lordosis after laminoplasty [30,32], and if their preservation is also connected with postoperative pain outcomes, then potentially C3 laminoplasty can be performed with minimal disruption of these attachment points. This is important to test, because in some patients the additional decompression provided by C3 open-door plated laminoplasty could be required. In our opinion, proper execution of C3 laminoplasty does not exclude preservation of C2 paraspinal muscle attachments.

## 5. Limitations

Limitations of the study are similar to those inherent to any retrospective study, which include the potential for selection bias. Specifically, patients whose treatment included a laminectomy may have a different pattern of degenerative cervical diseases, as compared to the laminoplasty-only cohort. Additionally, it should be noted that the “dome” laminectomy procedure requires additional technical expertise and may have variable effectiveness depending on surgeon experience with this procedure, which could explain differences in outcomes when comparing different surgical approaches. Surgeon experience with both procedures was balanced between groups in our single-institution study; however, subtle differences may still have had an impact, and further comparative studies could help elucidate the impact of surgeon experience on the effectiveness of C3 “dome” laminectomy at the rostral end of C4–C6 laminoplasty constructs. The laminoplasty-only cohort was characterized by an older age, which was mitigated by controlling for age in the multivariable ordinal regression. All cases were performed at a single center, so the disease prevalence and practice patterns at our tertiary academic center may lack external validity to the general community of spine surgeons. Although this cohort study was the first to include a comparator group when assessing the change in cervical lordosis following different surgical technique at the top level of a multilevel laminoplasty construct, the sample size of the cohort was limited. Like previous studies comparing surgical approaches in multilevel cervical laminoplasty, stringent inclusion/exclusion criteria allow for direct comparisons between more homogeneous groups at the expense of potentially identifying smaller changes in measured outcomes, such as the radiographic parameters, in this study. Our analysis was powered to detect clinically meaningful differences in the radiographic measures studied between groups both in the pre- and postoperative periods; however, given a larger sample size, smaller differences in preoperative radiographic measurements could have been detected. Therefore, the effect size of parameters such as loss of cervical lordosis should still be validated in other clinical settings to support the external generalizability of our presented results and unify expectations for changes following the procedures used in this study.

## 6. Conclusions

Loss of cervical lordosis of 5° or more following open-door cervical laminoplasty occurred in 31% of patients over a median follow-up of 24.2 months. There was 5–10° lordosis loss in 13% and greater than 10° lordosis loss in 18%. The surgical technique at the rostral C3 level (laminoplasty versus partial “dome” laminectomy) did not significantly influence loss of cervical lordosis following laminoplasty.

## Figures and Tables

**Table 1:** Demographic, Surgical, and Radiographic Data.

	Laminoplasty Only N = 61	+Partial C3 Laminectomy N = 39	*p* Value
Male gender	43 (70.5%)	21 (53.9%)	0.091
Age	66.4 ± 1.3	59.4 ± 2.3	**0.012**
Height (cm)	170.3 ± 1.4	169.5 ± 1.5	0.349
Weight (kg)	82.4 ± 2.4	81.8 ± 2.5	0.870
Body Mass Index	28.5 ± 0.8	28.5 ± 0.7	0.983
Not current smoker	55 (94.8%)	34 (91.9%)	0.566
Diabetes	12 (20.0%)	4 (10.3%)	0.198
Coronary Artery Disease	8 (13.3%)	2 (5.1%)	0.186
Peripheral Vascular Disease	1 (1.67%)	0 (0.0%)	0.418
Chronic Kidney Disease	4 (6.7%)	1 (2.6%)	0.362
Osteoporosis	5 (8.3%)	2 (5.1%)	0.543
American Society of Anesthesiologists (ASA) Classification			
1	0 (0.0%)	2 (5.3%)	0.273
2	30 (63.8%)	22 (57.9%)	
3	17 (36.2%)	14 (36.8%)	
Median Number of Intervertebral Levels Decompressed [Interquartile Range]	4 [4, 4]	3 [3, 3]	**<0.001** *
Estimated Blood Loss (mL)	179.0 ± 24.3	142.0 ± 12.2	0.178
Operative Time (minutes)	134.0 ± 8.2	138.8 ± 5.1	0.619
Length of Stay (days)	4.6 ± 0.9	3.5 ± 0.2	0.346
Change in Cervical Lordosis			
No change (<5°)	40 (65.6%)	29 (74.3%)	0.644
Mild change (5–10°)	9 (14.8%)	4 (10.3%)	
Moderate change (>10°)	12 (19.7%)	6 (15.4%)	
90-day Readmission Rate	1 (1.6%)	2 (5.1%)	0.318
1-year Reoperation Rate	0	1 (2.5%)	0.209
Median Follow-up [Range], months	26.5 months [12.2 months–9.0 years]	15.3 months [12.1 months–6.4 years]	**0.002**

*: The number of interspaces decompressed will be invariably higher in the +partial C3 laminectomy cohort because the top and bottom of the laminoplasty bed included a “dome” laminectomy. All bolded *p* values were statistically significant.

**Table 2:** Radiographic Measurements.

	Laminoplasty Only	+Partial C3 Laminectomy	*p* Value
Preoperative Measurements			
Cervical Lordosis	13.2° ± 1.2	11.1° ± 1.3	0.259
T1 Slope	32.9° ± 1.3	29.3° ± 1.6	0.073
T1 Slope–Cervical Lordosis	19.9° ± 1.2	18.6° ± 1.1	0.485
Cervical–Sagittal Vertical Axis (mm)	31.5 ± 2.1	27.4 ± 2.1	0.193
Global Measurements			
Pelvic Incidence	54.4° ± 2.2	60.1° ± 2.3	0.085
Pelvic Tilt	21.1° ± 1.7	19.5° ± 1.7	0.503
Lumbar Lordosis	43.5° ± 2.9	52.4° ± 3.4	0.056
Sagittal Vertical Axis (mm)	28.9 ± 7.1	30.4 ± 7.0	0.891
Postoperative Measurements			
Cervical Lordosis	9.4° ± 1.4	11.2° ± 1.2	0.331
T1 Slope	31.2° ± 1.2	27.8° ± 1.6	0.080
T1 Slope–Cervical Lordosis	21.7° ± 1.6	18.2° ± 1.5	0.126
Cervical–Sagittal Vertical Axis (mm)	34.0 ± 2.0	36.2 ± 2.4	0.480

**Table 3:** Ordinal regression for change in cervical lordosis: no change (<5°), mild change (5–10°), moderate change (>10°).

	Adjusted Odds Ratio [95% Confidence Interval]	*p* Value
Partial C3 “Dome” Laminectomy	0.72 [0.29–1.8]	0.479
Age	1.01 [0.97–1.05]	0.593

## Data Availability

The data presented in this study are available on request from the corresponding author. The data are not publicly available due to respect for patient privacy.
